# Visual cues do not enhance sea lion pups’ response to multimodal maternal cues

**DOI:** 10.1038/s41598-018-28171-w

**Published:** 2018-06-29

**Authors:** Kaja Wierucka, Isabelle Charrier, Robert Harcourt, Benjamin J. Pitcher

**Affiliations:** 1 Department of Biological Sciences, Macquarie University, Sydney, 2109 NSW Australia; 2 Institut des Neurosciences Paris-Saclay, Université Paris-Saclay, CNRS (UMR 9197), Université Paris-Sud, Orsay, 91405 France; 3 Taronga Conservation Society Australia, Mosman, 2088 NSW Australia

## Abstract

Mammals use multiple sensory cues for mother-offspring recognition. While the role of single sensory cues has been well studied, we lack information about how multiple cues produced by mothers are integrated by their offspring. Knowing that Australian sea lion (*Neophoca cinerea*) pups recognise their mother’s calls, we first tested whether visual cues are used by pups to discriminate between conspecifics of different age classes (adult female vs pup). We then examined if adding a visual stimulus to an acoustic cue enhances vocal responsiveness of Australian sea lion pups, by presenting wild individuals with either a visual cue (female 3D-model), an acoustic cue (mother’s call), or both simultaneously, and observing their reaction. We showed that visual cues can be used by pups to distinguish adult females from other individuals, however we found no enhancement effect of these cues on the response in a multimodal scenario. Audio-only cues prompted a similar reaction to audio-visual cues that was significantly stronger than pup response to visual-only cues. Our results suggest that visual cues are dominated by acoustic cues and that pups rely on the latter in mother recognition.

## Introduction

Animal communication can be extremely complex and may use multiple sensory modalities^1^. Due to differences in environmental conditions, cue structure and limitations of sensory systems themselves, the costs and benefits of conveying information through each modality vary^2^. Accordingly, animals often invoke multiple sensory modalities simultaneously, presumably to increase the robustness or diversity of transmitted information^2–7^. Multimodal communication occurs when composite signals or cues are received through more than one sensory channel^4,5,8^. When multiple cues are present, they may be redundant, i.e. conveying multiple copies of the same information, or non-redundant, i.e. conveying multiple, different messages thereby enabling the transmission of more information^8^. Furthermore, when combined, cues may induce different responses from the receiver^8^. Multiple redundant cues may elicit either an equivalent or an enhanced response compared to a single cue, and non-redundant cues may be independent, cause dominance or modulation, or lead to the emergence of a new response^8^. Because of these interactions, investigating how animals respond to multiple cues simultaneously is necessary, as it provides greater understanding about complex behaviour than simply looking at cues in isolation. However, cues may be used and integrated differently by animals, depending on the interactions between the costs and benefits of obtaining them^9^. Given there are limitations for specific cues to convey information (e.g. transmission distance) and costs associated with multisensory signal production and perception, using multiple cues is not always favoured^9^. Determining why and when animals use specific combinations of cues, gives insight into the intricacies of multimodal communication. To understand how communication systems evolved and what rules they are governed by, the interactions and relevant importance of sensory cues in a given context need to be explored.

Mother-offspring recognition is known to involve different and usually multiple modalities simultaneously, with acoustic, olfactory and visual cues playing varying roles for different mammalian species^10,11^. The ability to identify young by mothers and its reciprocal is usually beneficial to both parties. It allows mothers to direct their care only towards filial offspring thereby enhancing potential reproductive output^12^. For offspring, identifying their mother may limit the risk of injury caused by approaching non-kin and limit energy wasted in unsuccessful begging attempts^12^. While extensive literature has investigated mother-young recognition abilities of many mammalian species^13–18^, most studies have investigated only the role of single sensory modalities. Under natural conditions, sensory cues co-occur and will rarely be available for inspection in isolation, yet there is not much information about the combined effect of different cues on the response of the receiver. No wild studies exist, although there is some evidence from domestic and laboratory mammals on the relative importance of individual sensory cues used in concert by mother and offspring sheep (*Ovis aries*)^19–27^, goats (*Capra hircus*)^28^ mice (*Mus musculus*)^29^ and rats (*Ratus norvegicus*)^30^. While a vast majority of these studies focus on recognition of the offspring by mothers, it is likely that mothers and offspring utilise cues differently, as apart from discrimination abilities, their motivation and therefore costs and benefits of obtaining cues are very different.

Mother-offspring recognition is especially important for colonial mammals with mobile young and frequent mother-offspring separations occurring due to the mother needing to leave periodically to forage, such as fur seals and sea lions^31^. Acoustic, olfactory and visual cues are all used in the mother-pup reunion process^18^. For different otariid species, a similar pattern of the reunion has been observed – the female and pup call to each other, they look for each other, and when at close range nasal investigations are performed^31^. Although observational studies exist for multiple species^32–36^ extensive experimental work about recognition through different sensory modalities has been done only for the Australian sea lion (*Neophoca cinerea*). Previous research demonstrated that both pups and adults produce individually stereotyped calls^37^, and females use acoustic^38–40^, olfactory^41^ and visual^42^ cues to recognise filial pups. Vocal recognition is mutual as pups can accurately distinguish their mother’s calls from that of other females^40^, yet the onset of this ability is delayed compared to mothers^43^. Although information is available for pup recognition by females, what role non-vocal cues play, and the interaction between cues, in the pups’ recognition abilities of mothers remains to be evaluated. Australian sea lions provide a unique opportunity to look at the role of the receivers’ costs in shaping recognition systems. The main constraints for the use of multimodal cues are perception and production costs of cues, as well as the risk of increased eavesdropping and therefore higher detection rates by predators^4^. This species does not have terrestrial predators and the cost of producing cues is negligible. Therefore, in a situation where cues are capable of conveying useful information, the only limitations for using multimodal cues are the costs of obtaining, receiving, processing and integrating cues, and their survivorship consequences^4^.

In this study we first test whether visual cues can be used by pups to discriminate among conspecifics (adult females vs pups) and then examine whether visual and acoustic cues induce a synergistic effect on the behavioural response of Australian sea lion pups during mother-pup reunion.

## Methods

### Study site and animals

The use of visual cues by pups (visual experiment) was studied in a wild population of Australian sea lions inhabiting Olive Island (32°43′S, 133°58′E) and Kangaroo Island (35°59′S, 137°19′E) in April and October 2016, respectively. Experiments examining multimodal cue use by pups (bimodal experiment) were conducted in September-October 2017 on Olive Island. Pups used in both experiments were less than 4 months old and were approached for procedures when mothers were away on foraging trips, to avoid mother-pup separation and thus limit disturbance. Pups used in the bimodal experiment (only 2–4 month olds) were captured and restrained for a short period of time where they were individually marked by clipping a unique symbol into their fur and applying hair dye (Clairol Nice’n Easy©). This allowed us to identify pups at a distance without the need to approach them and to identify their mother in order to record their pup attraction calls.

### Sample collection

Pup attraction calls were recorded from mothers of marked pups during interactions with their pups in the colony using a BeyerDynamic M69 TG microphone (frequency response: 50Hz–16 kHz ± 2.5 dB; BeyerDynamic, Heilbronn, Germany) mounted on a 3 m boom connected to a Marantz PMD 671 digital recorder (Marantz Europe, Eindhoven, Netherlands). Calls were recorded at a 44.1 kHz sampling frequency. Good quality calls (i.e., no background noise and no overlap with other vocalizing animals) were selected and high-pass filtered at 200 Hz using Avisoft SAS Lab Pro (Avisoft Bioacoustics, R. Specht) to remove low frequency noise caused by wind and/or waves. Experimental playback series were composed of six calls separated by 2–3 seconds of silence, similar to a natural calling sequence of a female searching for her pup. The playback series were broadcast using a portable amplified speaker (JBL Flip 3, 2 × 8 W, frequency response: 85Hz-20 kHz) connected by Bluetooth to an audio player. Calls were played at an approximately natural amplitude of 83 ± 3 dB SPL measured 1 meter from the source^40,43^.

**Figure 1:**
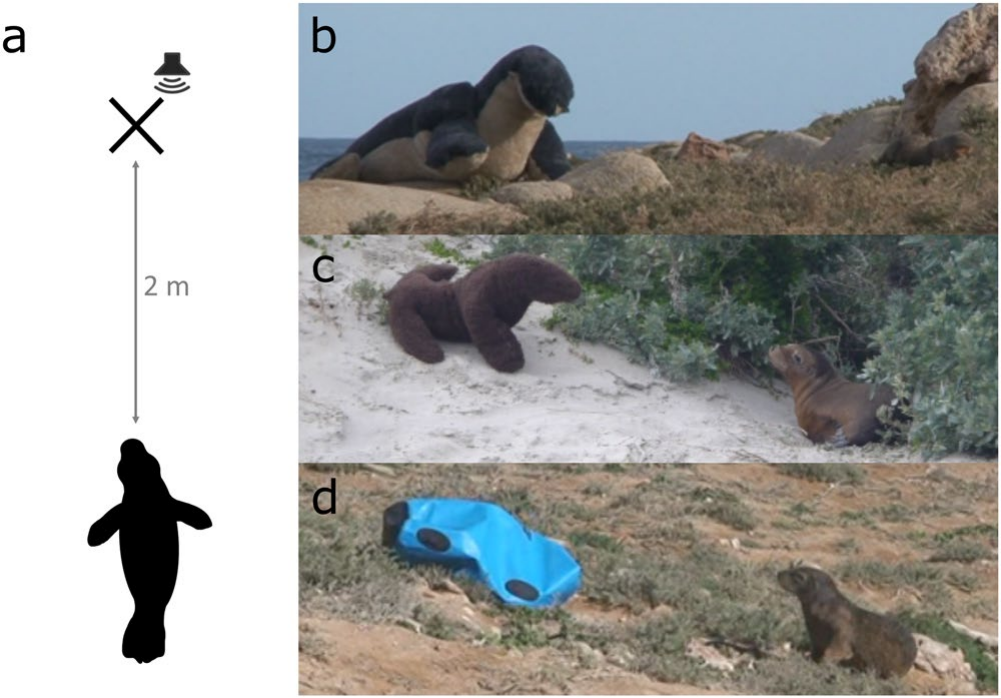
Behavioural experiment setup (**a**). Pups were presented with a stimulus (marked with ‘x’) - either a female model (**b**), pup a model (**c**), or foreign object (**c**), placed 2 meters away from the animal, directly in line of sight, facing toward the individual. The speaker (where applicable) was placed adjacent to the model. When the visual model was absent (audio-only cues), the speaker was present in the same location.

3D-models imitating an adult female as well as a 1–2 and a 2–4 month old pup were constructed using synthetic fur with polyester filling, and fitted with a wire skeleton to maintain an upright posture (Fig. 1). To examine the role of class-level visual cues in recognition, the size and fur colour pattern were chosen based on the average body size and colouration of adult females as well as 1–2 and 2–4 month old pups (body length: 156, 76 and 87 cm respectively). As all animals within a given age/sex class appear similar and no information currently exists about whether individual visual recognition is possible in pinnipeds, models approximated the size, shape and colour brightness of the respective age/sex groups. Our pup models have been previously shown successful in imitating animals for research purposes^42^.

### Experimental design

In both the visual and bimodal experiments, the stimulus was presented 2 meters away from the pup, facing the pup’s head or at least within 45° to ensure a clear visibility of the model presented (Fig. 1). The models did not contain any sea lion olfactory cues, and were placed beyond the range that olfactory assessment appears to occur^41^. Objects were placed while the pup was sleeping as to not disrupt, startle or otherwise confound the response of the pup with human presence. The pup was woken up with a natural sound (i.e., a hand clap) immediately prior to presentations.

For the visual experiment, 25 pups were presented with one of three treatments: (1) female model – a life-size model of an adult female sea lion (n = 8;); (2) pup model – a life-size model of pup of the same age as the tested pup (n = 9); (3) control – a foreign object (i.e a 65 L blue dry bag filled with air; n = 8). We expected pups to be vigilant if they identified the presented object as a female as non-related females are aggressive towards non-filial pups^32,44,45^. We also predicted that pups would not change their location and return to their initial behaviour if they identified the presented object as another pup, as pups associate with each other in the colony during maternal foraging trips on a regular basis and pose no threat to each other^32^. Therefore, a significant difference in pup response to different models would indicate the use of visual cues for conspecific assessment, whereas no differences would point to the adult female models not being identified as non-mothers, and the possibility that visual cues are not used by pups. Based on this, an ethological scale was created to assess whether the pups could distinguish different categories of conspecifics/items based solely on visual cues. The behaviour of the pup following it looking at the object was scored and two patterns were defined: “return to rest” – when no change in location occurred and the pup returned to its initial resting position following the presentation, and “vigilance” – when the pup moved away from the object, or stayed in the general area without returning to a resting position.

During the bimodal experiment, one of three treatments was presented to 30 pups: (1) audio – pup attraction calls of their mother (n = 10); (2) visual – the life-size model of an adult female sea lion (n = 10); (3) audio-visual – pup attraction calls of their mother paired with the life-size adult female model (n = 10). As we were measuring whether there is an enhancement effect following the addition of the visual cue to the acoustic cue, we noted the number of calls produced by the pup as well as the latency to call (if a call occurred) during 60 seconds after the beginning of each presentation.

### Statistical analysis

A Fisher’s exact test, with Holm’s correction for multiple comparisons was used to assess whether differences in response among treatments occurred in the visual experiments. The number of calls produced by pups among treatments in the bimodal experiment were compared using a Kruskal-Wallis test with a Dunn’s post-hoc test^46^. An exact Wilcoxon rank sum test was used to examine differences in latency to call between audio and audio-visual treatments^47^. All statistical analyses were performed in R version 3.2.2^48^.

The research was carried out under the permission of the South Australian Wildlife Ethics Committee (approval 30/2015) and the Department of Environment, Water and Natural Resources (permit E26447). All experimental procedures followed the Australian code of practice for the care and use of animals for scientific purposes. All data analysed during this study are included in this published article (and its Supplementary Information files).

## Results

**Figure 2:**
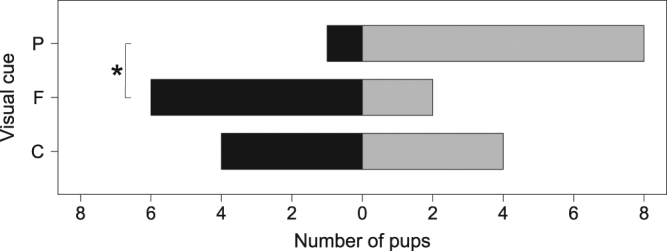
Number of pups returning to a resting state (grey) and displaying vigilance (black) in response to different presented visual stimuli. Notations: P – pup model, F – female model, C – control. The asterisk indicates statistically significant differences (p = 0.046) among treatments.

Pup response varied depending on the presented visual cue (p = 0.031). Pairwise comparisons showed that this was due to a significant difference between pup responses to the female and pup models (female model vs pup model: p = 0.046, control vs pup model: p = 0.262; control vs female model: p = 0.608, Fig. 2). Eight out of nine pups presented with a pup model returned to a resting state after looking at the object. In contrast, six out of eight pups that were shown the female model responded to the treatment with vigilance. Pup response to the control varied, with half of the tested pups returning to a resting position and half staying alert or moving away from the object.

**Figure 3:**
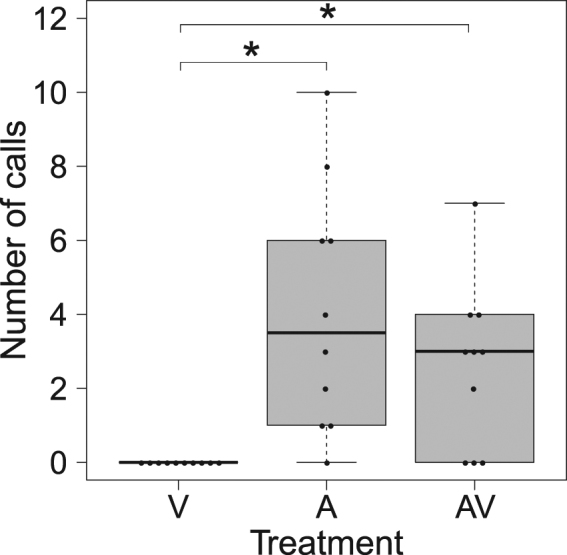
Number of calls produced by pups during visual-only (V), audio-only (A) and audio-visual (AV) treatments. Boxplots show the median, quartiles and minimum and maximum values within the inter-quartile range. Asterisks indicate statistically significant differences (V vs A p = 0.0007, V vs AV p = 0.01) among treatments.

**Figure 4:**
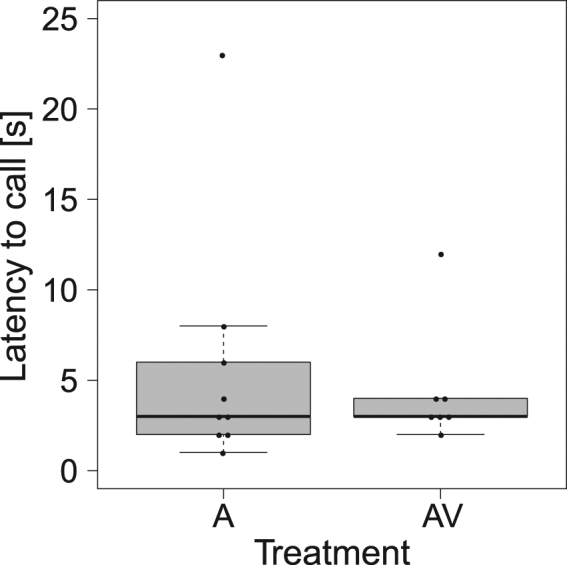
Pup latency to call during audio-only (A) and audio-visual (AV) treatments. Boxplots show the median, quartiles, and minimum and maximum values within the inter-quartile range. No significant difference was found between treatments.

When exploring bimodal cue use, we found significant differences in the number of calls produced among treatments (χ^2^ = 14.72, df = 2, p = 0.0006; Fig. 3). The audio and audio-visual presentations elicited a statistically similar response that was significantly different from that exhibited during visual-only treatments (Dunn’s test: visual vs audio: p = 0.0007; visual vs audio-visual: p = 0.01; audio vs audio-visual: p = 0.35). Seven out of ten pups produced calls following audio-visual presentations, nine out of ten pups exposed to acoustic-only presentations responded vocally to the playback, and none of the animals presented with just the visual treatment produced calls. Furthermore, we found no significant differences in the latency to call between audio-only and audio-visual presentations (W = 30, p = 0.95, n = 16; Fig. 4).

## Discussion

We showed that visual cues can be used by sea lion pups to distinguish between pups and adult females. However, we found no enhancement of the pups’ response in the presence of multiple cues, with combined audio-visual cues having the same effect as audio-only presentations, and both prompting a stronger response than the visual-only treatment. Our findings demonstrate that class-level visual cues (i.e., indicating an adult female) are dominated by acoustic cues, and are not used by pups to facilitate reunion, with offspring relying mainly on information conveyed in acoustic cues for mother recognition.

Australian sea lion pups showed pronounced differences in response when presented with a range of objects that either simulated conspecifics or were not biologically relevant and were able to distinguish adult female morphs from pup morphs based solely on visual cues. Pups displayed vigilance when presented with female models, but returned to a resting state following pup model presentations. When presented with the control which had no biologically relevant cues, but which was novel, there was no distinct response, with pups either responding with vigilance or without in equal measure. Overall this clearly demonstrated that pups can visually distinguish broad age classes of conspecifics. Our visual models were indicative of an adult female and did not provide any cues that might be used for individual recognition. Yet overwhelmingly (75%) pups responded as they would to an unfamiliar female, with vigilance. Our experiment is the first step towards understanding information perceived by pups through visual cues. Although we were unable to test individual visual recognition, we demonstrate that visual cues provide a broad assessment of animals at least to a given sex/age class and to the presence/absence of an animal in close proximity to the receiver.

Being able to identify the correct age/sex class of an individual using broad-brush cues may be beneficial when attempting to find a specific individual within a colony, as it refines the search to a subset of animals. Australian sea lion pups produce more calls in response to calls of their mothers compared to that of other females^43^. We thus expected them to further increase call rates once a potential mother is within sight, as it would allow them limit energetic expenditure by increasing call rates only when chances of reunion are higher or decrease call rates if the model was visually identified as being non-mother. However, pups tested in our study showed no enhancement in behavioural response when presented with multimodal cues compared to unimodal ones. Pups produced a similar number of calls to the audio/visual stimulus as to the audio-only stimulus, with no vocal reaction to the visual-only cues. The absence of enhancement points to a lack of interaction between acoustic and visual cues and the pups’ lack of use of class-level visual cues when identifying their mothers. Based on the response of pups to female models when testing the role of visual cues, we ruled out the possibility that the absence of enhancement was simply a result of the pups identifying the female models as non-mothers. In our experiment, pups showed vigilance when presented with female models. If the model was identified as a non-mother in the bimodal experiments, we would have expected a decrease in call production, which was not the case. Ruiz-Miranda^28^ suggested that for goat kids, visual cues are more important than acoustic and olfactory cues. Only broad cues were tested (pelage colour) while acoustic cues were individually distinctive and olfactory cues were masked. Although the tested visual cues contained only broad information, they were of higher importance than individually distinctive acoustic cues, thus showing that even when broad, visual cues have the ability to induce increased response. In our study, adding the visual cue did not change the pups’ response, therefore the most parsimonious explanation is that while pups are capable of differentiating classes of individuals based on visual cues, they do not use class-level visual cues in a multimodal context, suggesting the presence of other factors that limit the use of both cues simultaneously.

The active space of cues varies as a function of the characteristics of a given cue, its production and perception, as well as the environment through which it travels^2,49,50^. Acoustic cues are generally considered to function at long range and visual cues are classified as mid to short range cues^50^. Differences in cue active space are regarded to be one of the main factors favouring multimodal communication^50^. However, for otariid pups, the differences in active space of sensory cues are important in context of risk of injury, as females can be extremely aggressive towards non-filial pups that approach them^44,45,51^. In this case, differences in active space could limit the use of multiple modalities, as cues with a smaller range may require pups to come out of hiding and become exposed to getting attacked or trampled by other individuals, or if they approach an individual to obtain useful information it may put them at risk of injury. Consequently, it seems that pups rely on hearing – the one modality that allows them to acquire accurate and reliable information at long range^39,40^ for the assessment of female identity prior to approach.

Munoz and Blumstein^9^ proposed a framework within which there is a plausible explanation for the evolution of bimodal responses, from the cost-benefit perspective of the receiver. The authors define three predictions for multisensory integration: enhancement – when the costs of missing information are high and outweigh the costs of obtaining cues; antagonism – when combined cues point to a lower likelihood of an event; and equivalence/dominance – when obtaining more information is too costly and therefore multimodal cues are not used^9^. This framework may help explain why pups do not combine acoustic and visual cues, and the evolutionary significance of this choice. We found the pup response to the bimodal and acoustic presentations to be the same or higher than the response to visual-only cues, and from our visual experiment we know that visual cues can be used in age-class conspecific assessment. Accordingly, our results fit the equivalence/dominance scenario, suggesting that acquiring information conveyed in visual cues does not outweigh the cost of obtaining them. This might be due to the risks of obtaining useful information being high or to the information encoded within them not providing any more useful information than the acoustic cues. The evolutionary pressures and mechanisms for this scenario to evolve could be investigated in more detail. However, regardless of which explanation plays a larger role, our findings indicate that the costs associated with obtaining information limit the use of multimodal cues in mother recognition by pups, with the characteristics of female-pup interactions as well as the consequences of differences in cue active space discussed above, also supporting this argument.

We have demonstrated that although Australian sea lion pups have the ability to use visual cues for conspecific assessment, they are not used in a multimodal context and are dominated by acoustic cues. By allowing the offspring to obtain detailed information at a distance, the use of acoustic cues does not entail a risk of injury from non-mother females and provides a stable and reliable way of mother identification on their own. Although reliance on a single modality may be disadvantageous^8^, we show that using cues in a multimodal context is not always beneficial, even when the risk of increased predation caused by eavesdropping and cue production costs are low or absent. The cost-benefit ratio of obtaining information seem to play a significant role in limiting the use of multimodal cues and this role in the evolution of communication systems should be examined in more detail.

## Electronic supplementary material

Supplementary Dataset
